# An Injectable Fibrin Scaffold Rich in Growth Factors for Skin Repair

**DOI:** 10.1155/2021/8094932

**Published:** 2021-02-08

**Authors:** Zhengwei Shao, Chengqi Lyu, Lin Teng, Xuetao Xie, Jiayue Sun, Derong Zou, Jiayu Lu

**Affiliations:** ^1^Department of Stomatology, Shanghai Jiao Tong University Affiliated Sixth People's Hospital, Shanghai 200233, China; ^2^School of Chemistry and Chemical Engineering, Shanghai Key Laboratory of Electrical Insulation and Thermal Aging, Shanghai Jiao Tong University, Shanghai 200240, China; ^3^Department of Orthopaedic Surgery, Shanghai Jiao Tong University Affiliated Sixth People's Hospital, Shanghai 200233, China; ^4^Shanghai Key Laboratory of Sleep Disordered Breathing, Shanghai Jiao Tong University Affiliated Sixth People's Hospital, Shanghai, China

## Abstract

Platelet aggregates, such as PRP, PRF, and CGF, have been used alone or in combination with other grafting materials to enhance restoration outcomes. The process for preparing these autografting materials requires two-step centrifugation or specific centrifuges. In this study, we obtained an injectable fibrin scaffold (IFS) rich in growth factors by one-step centrifugation of whole blood from rabbits. The purpose of this study is to introduce some characteristics of IFS. This scaffold was characterized using various techniques, including Masson's trichrome staining, scanning electron microscopy, porosity measurements, and cell counting. The sustained release of growth factors, including PDGF, VEGF, TGF-*β*1, IGF, FGF, and EGF, was quantified using ELISA assay. The obtained IFS was tested for its effects on cell proliferation, extracellular matrix deposition, and full-thickness skin defect repair. The prepared IFS is characterized by a loose fibrin network structure with white blood cells and platelets that slowly release growth factors and can promote the healing of skin defects via the promotion of cell proliferation, collagen deposition, and tissue revascularization. In addition, its liquid properties and porous structure are conducive to its application as a therapeutic component in tissue engineering.

## 1. Introduction

Growth factors are an important component of tissue engineering as they can provide a large number of biological signaling molecules to a given microenvironment, mobilize seed cells and peripheral cells in vivo to adapt quickly to the implantation bed's environment, and activate tissue repair [1, 2]. The use of autologous platelet concentrations excludes the possibility of immunologic rejection and cross-infection of exogenous growth factors [3, 4]. Platelet-rich plasma (PRP), which represents the first generation of autologous blood-based concentrates, is rich in growth factors and can be obtained using a double centrifugation process. PRP contains a high concentration of platelets that can secrete a high volume of growth factors to promote tissue regeneration and repair [5]. However, various concerns have been raised regarding the addition of anticoagulants and bovine serum that call for more feasible strategies.

With the aim of improving and streamlining the preparation methods, platelet-rich fibrin (PRF) was developed as a source of autogenous blood-derived growth factor concentrate that could be prepared without the addition of thrombin [6–8]. In the field of stomatology, PRF has been used to reduce bleeding after a tooth extraction [9], relieve osteitis symptoms [10], improve the quality of healing in a socket after a tooth extraction [11], and treat gingival recession [12], etc. Recently, a new protocol for the preparation of PRF has been formulated, in which the centrifugation procedures have been modified. While the standard for PRF preparation is to centrifuge at 2700 rpm for 12 min, Choukroun, one of the original developers of this method for isolating PRF, altered the preparation procedure to yield advanced platelet-rich fibrin (A-PRF). A-PRF is centrifuged at 1500 rpm for 14 min and has been shown to contain a greater number of platelets and monocytes/macrophages [13]. Concentrated growth factors (CGF) are another modified form of PRF. CGF are prepared by repeatedly switching the centrifugation speed and have been characterized as relatively rigid fibrin clots [8]. The fibrin matrixes of PRF, A-PRF, and CGF contain high concentrations of growth factors, leucocytes, and platelets, which enable them to provide favorable microenvironments for cell migration and sustained release of growth factors for cell proliferation and differentiation [4]. Consequently, they have the potential to promote the regeneration of soft and hard tissues [14]. Furthermore, component immune cells provide them with the ability to regulate inflammation and anti-infection activity [4]. Due to the absence of anticoagulants, the consistency fibrin scaffold in PRF, A-PRF, and CGF inadequates combined with bone grafts.

A liquid formulation can either be utilized alone or in combination with various biomaterials. A liquid formulation of platelet-rich fibrin, termed injectable-PRF (i-PRF), has been investigated using centrifugation at 700 rpm for 3 min (60 g) without anticoagulants [15]. Data suggest that i-PRF can accelerate the proliferation of fibroblasts. Meanwhile, we obtained an injectable fibrin scaffold (IFS) rich in growth factors by centrifugation at 3000 rpm for 10 min. This liquid scaffold contains large amounts of fibrins, which trap a large number of platelets, white blood cells, and growth factors, and can promote cell proliferation, cell migration, and matrix secretion. The IFS can promote the secretion type I and type III collagens by skin fibroblasts to promote in vivo skin repair. We also examined the mechanisms by which the IFS promotes skin repair, with the results suggesting that the IFS promotes tissue repair by regulating *MMP-1*, *MMP-9*, *TIMP-1*, and *TIMP-2*. In addition, the sparse fibrin network in the liquid scaffold is conducive to both the stable and sustained release of growth factors for more than two weeks in addition to carrying seed cells. Hence, this IFS can be used in regenerative medicine either alone or as a supplement to other biomaterials.

## 2. Materials and Methods

### 2.1. Preparation of the IFS

All animal experiments were approved by the Animal Welfare Ethics Committee of the Shanghai Sixth People's Hospital affiliated with the Shanghai Jiao Tong University School of Medicine (DWSY2019-024). After being anesthetized intravenously with pentobarbital sodium (30 mg/kg), the chest hair of adult rabbits (12 months old, average weight 2.5 kg) was removed, and the skin was sterilized. Then, 10 mL of whole blood was extracted from the heart and transferred into a centrifuge tube containing anticoagulant (heparin lithium). After centrifugation at 3000 rpm for 10 min (TR-18, Trausiam, Changzhou, China), 1 mL of transparent liquid (named IFS) was collected from 3 mm above the junction point of the erythrocyte aggregation at the bottom of the tube. Penicillin (800,000 U) was injected to prevent infection.

### 2.2. Characterization of IFS

#### 2.2.1. Masson Staining of IFS

A drop of IFS was smeared on a slide. After drying with air, the smear was stained with Masson's trichrome staining. A histological analysis was performed according to the kit's instructions.

#### 2.2.2. SEM of IFS

SEM imaging and analysis were conducted to evaluate the pore morphology of IFS. After being fixed in 2.5% glutaraldehyde for 24 h at 4°C, an IFS sample was processed through graded concentrations of tert-butanol. Then, the sample was gently washed with phosphate-buffered saline (PBS), quick-frozen at −80°C for 12 h, and lyophilized in a freeze-dryer (ALPHA 1-4 LDplus, Martin Christ, Osterode, Germany) under vacuum for 48 h. The specimens were cross-sectioned and sputter-coated with gold. The morphology of the IFS sample was observed by Scanning electron microscopy (Phenom Pro, Eindhoven, Netherlands).

#### 2.2.3. Specific Surface Area of IFS Pores

The pore characteristics of the IFS were measured using an advanced micropore size and chemisorption analyzer (ASAP 2460, Micromeritics Instrument Corp, Norcross, GA, USA). The sample was outgassed, first at room temperature and then at 623 K, to a pressure of <0.2 Pa. The specific surface areas were determined using the nitrogen adsorption values from *P*/*P*0 = 0.057–0.294 via the BET method. The microporous pore volume was determined from the amount of adsorbed N_2_.

#### 2.2.4. Complete Blood Count

A complete blood count was conducted using an automatic hematology analyzer (Sysmex XS-800i, Japan). Uncentrifuged rabbit heart blood was used as a control. The experiments were repeated five times.

#### 2.2.5. Sustained Release of Growth Factors

One milliliter (1 mL) of IFS was added to 2 mL of PBS and incubated at 37°C to allow growth factor release into PBS. The supernatant was collected at different time points (0, 1, 2, 3, 4, 5, 6, 9, 12, and 15 days). The sustained release of growth factors, including PDGF, VEGF, TGF-*β*1, IGF, FGF, and EGF, was quantified using ELISA assay according to the manufacturer's protocol. Briefly, 40 *μ*L of assay diluent and 10 *μ*L of sample were coincubated for 30 min at 37°C in 96-well plates precoated with antibody. Wells were washed five times with washing buffer and incubated for 30 min with peroxidase-conjugated antibody solution. Enzyme substrate solution was then added. After 15 min of incubation in darkness, the enzyme reaction was stopped by adding 50 *μ*L of stopping solution. Absorbance was measured at 450 nm on a microplate reader (iMark, Bio-Rad, Hercules, CA, USA). All samples were measured in triplicate. All ELISAs used in this study were specific for rabbit.

### 2.3. Isolation and Culture of Rabbit Bone Marrow Stromal Cells (BMSCs) and Skin Fibroblasts (SFs)

To isolate BMSCs, 5 mL of bone marrow was harvested from the iliac crests of rabbits and cultured in Dulbecco's modified Eagle's medium (DMEM) (Hyclone, Thermo Fisher Scientific, USA) containing 10% FBS (Thermo Fisher Scientific, USA), 100 U/mL penicillin, and 100 U/mL streptomycin at 37°C in a 5% CO_2_ incubator (Heracell VIOS 160i, Thermo Scientific, Waltham, MA, USA). The culture medium was changed every 2 days. The cells were passaged when they reached a confluence of 80%.

To obtain SFs, a piece of full-thickness skin with dimensions of approximately 1 cm × 3 cm was carefully dissected from the neck of each rabbit. Subcutaneous adipose tissue and epidermis were carefully removed. The dissected tissue was cut into 1 mm × 1 mm pieces and digested with 0.2% type I collagenase at 37°C for 4 h. After digestion, a cell suspension was seeded on a cell culture plate and cultured at 37°C in a 5% CO_2_ incubator. The culture medium was changed every 2 days. The cells were also passaged when they reached 80% confluence.

### 2.4. MTT Assay of BMSCs and SFs

The proliferation of BMSCs and SFs in response to different concentrations of IFS was measured using MTT assay. BMSCs and SFs were seeded in 96-well plates (8000 cells/well). After 24 h of attachment, cells were treated with different concentrations of IFS (0%, 1%, 5%, and 10% for volume ratio) for 48 h. Twenty microliters (20 *μ*L) of MTT solution (5 mg/mL) was added to the wells and coincubated in the dark at 37°C in 5% CO_2_ for 4 h. Then, the media was removed and 150 *μ*L of DMSO was added to dissolve the formazan. After 10 min of gentle horizontal shaking, the cell viability was assessed by measuring the optical density (OD) at 490 nm in a full spectrum microplate reader (iMark, Bio-Rad, USA). All experimental groups and controls were run in triplicate.

### 2.5. Collagen and Collagenase Secretion Assay of BMSCs and SFs

BMSCs and SFs were seeded in 24-well plates (5 × 10^4^ cells/well). After 24 h of attachment, cells were treated with different concentrations of IFS (0%, 1%, 5%, and 10% for volume ratio) for 24, 48, and 72 h. The supernatant was collected to evaluate type I collagen, type III collagen, and type I collagenase secretion using ELISA assay as previously described.

### 2.6. Gene Expression

For real-time PCR experiments, SFs were seeded in 6-well plates (2.5 × 10^5^ cells/well). After 24 h of attachment, cells were treated with different concentrations of IFS (0%, 1%, 5%, and 10% for volume ratio) for 48 h. Whole RNA was extracted from SFs according to the manufacturer's protocol (Invitrogen, USA). A PrimeScript reverse reaction reagent kit (Thermo Fisher Scientific, USA) was used to carry out reverse transcription of RNA in accordance with the manufacturer's protocol. Quantitative analysis of the change in expression levels of *MMP-1*, *MMP-9*, *TIMP-1*, and *TIMP-2* genes was conducted using Maxima SYBR Green Master Mix (Thermo Fisher Scientific, USA) in an ABI PRISM 9700 PCR (ABI, USA). qPCR was performed at 94°C for 10 min and 40 cycles of amplification, which consisted of a denaturation step at 94°C for 15 s, an annealing step at 65°C for 30 s, and an extension step at 72°C for 30 s. The primer sequences used in the qPCR are shown in Table 1. The relative expressions of these genes were normalized to the expression of the *β*-actin gene. The change in expression of mRNA was assessed by the 2^−*ΔΔ*Ct^ approach.

### 2.7. Repair and Detection of Skin Defects

Six nude mice (8 weeks old, weighing 20 ± 3 g) were used to evaluate the repair of skin defects. Four full-thickness circular defects (5 mm in diameter) were created on the dorsum of each nude mouse. The skin defects were randomly covered with four materials (20 *μ*L IFS, 20 *μ*L IFS + 10 *μ*L SF suspension (5 × 10^5^/mL), 20 *μ*L normal saline (NS) + 10 *μ*L SF suspension (5 × 10^5^/mL), and 20 *μ*L NS as control). After surgery, all mice were housed separately and monitored daily. The defects in each individual nude mouse were photographed digitally on days 2, 4, and 7. The defect area was measured by tracing the wound's margin and calculated using NIH ImageJ 1.51 software.

### 2.8. Histological Study

For histological analysis, all mice were sacrificed 7 days after surgery. A circular section around the newly growing skin was harvested and fixed in 10% neutralized formalin. The specimens were then embedded in paraffin, vertically sectioned (5 *μ*m in thickness), and stained with hematoxylin–eosin (HE) and Masson's trichrome stain for histological evaluation. For immunohistochemistry staining, sections were incubated for 10 min with 3% hydrogen peroxide, incubated with 10% normal serum at 37°C for 30 min, and then incubated with primary antibodies against collagen I (PA5-29569, Thermo Fisher Scientific, USA) and VEGF (MA1-16629, Thermo Fisher Scientific, USA) at 4°C overnight. All sections were visualized using DAB (Sigma-Aldrich, St. Louis, MI, USA). Stained sections were examined using an Olympus light microscope (Olympus BX51, Tokyo, Japan).

### 2.9. Statistical Analysis

Data were analyzed using SPSS software (25th edition, IBM, New York, NY, USA). The values are expressed as means ± standard deviation (SD). Whenever appropriate, one-way ANOVA was used to discern the statistical difference between multiple groups. A probability value (*p*) of less than 0.05 was considered to be statistically significant.

## 3. Result

### 3.1. Acquisition of the IFS

**Figure 1:**
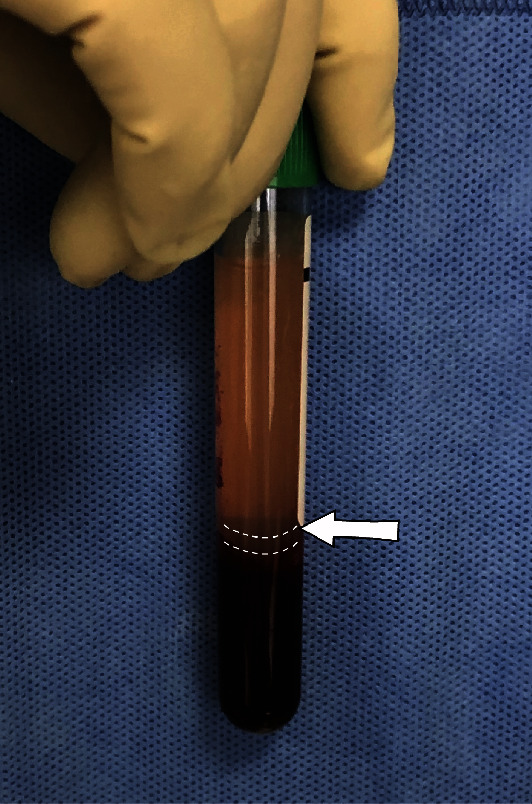
The IFS layer (between the white dotted lines) is obtained following whole-blood centrifugation.

Figure 1 shows, from bottom to top, the erythrocyte deposition layer, IFS layer, and then plasma layer in the test tube after centrifugation.

### 3.2. Characterization of the IFS

**Figure 2:**
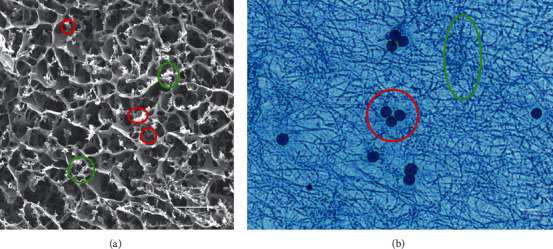
Characterization of the IFS. An SEM image (a) and Masson's trichrome staining (b) of the IFS show the porous scaffold structure in which white blood cells (red circles) and platelets (green circle) are trapped. Scale bars, 10 *μ*m.

Figure 2(a) shows an SEM image of the porous scaffold structure of the IFS. The pore size is 10 *μ*m, and the size of the interconnecting pores is 2–3 *μ*m. The porosity of a scaffold is important for allowing it to carry cells and growth factors. The BET surface area of the IFS is 16.3 ± 1.6 m^2^/g. Leukocytes and platelets can be seen to adhere to the pore wall and the edge of the fibrin scaffold network. Masson's trichrome staining of the IFS shows sparse fibrous reticular structures in which white blood cells and platelets are trapped (Figure 2(b)). The complete blood count shows that the IFS comprises approximately 25% white blood cells and nearly 50% of the platelets in whole blood. Specifically, 82.4% ± 11.3% of the white blood cells contained in the liquid scaffolds were lymphocytes.

### 3.3. Sustained Release of Growth Factors

**Figure 3:**
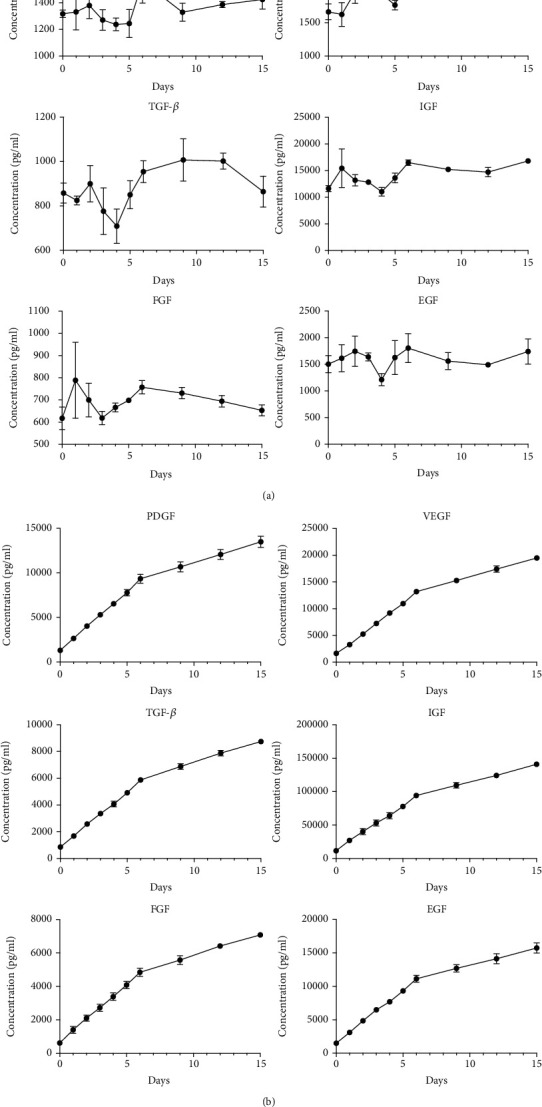
Sustained release of growth factors. Quantitative data on growth factors released at each time point (0, 1, 2, 3, 4, 5, 6, 9, 12, and 15 days) (a) and cumulative sustained release of growth factors (b).

The IFS showed sustained release of PDGF, VEGF, TGF-*β*, IGF, FGF, and EGF over the 15-day observation period (Figure 3). Specifically, the concentration of secreted growth factors decreased slightly after 4–5 days, then recovered and lasted for two weeks.

### 3.4. IFS Promotes the Proliferation of BMSCs and SFs

**Figure 4:**
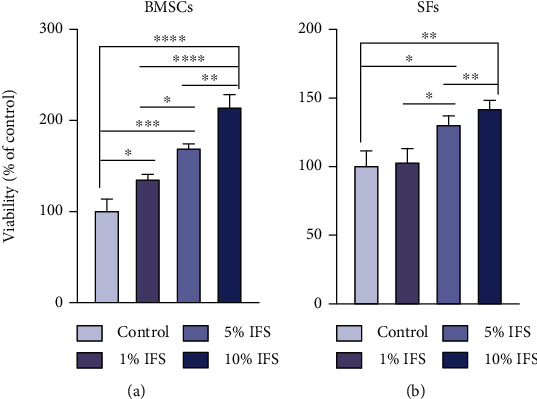
MTT assay of BMSCs and SFs treated with different concentrations of IFS for 48 h. (^∗^*p* < 0.05, ^∗∗^*p* < 0.01, ^∗∗∗^*p* < 0.005, and ^∗∗∗∗^*p* < 0.001 for the IFS-treated groups compared with the control group).

The MTT assay results show that the IFS could promote the proliferation of BMSCs and SFs after treatment for 48 h (Figure 4). After treatment with IFS for 48 h, the proliferative effect was found to be positively correlated with the concentration of IFS. As the concentration increased, the effect on proliferation became more significant.

### 3.5. Collagen and Collagenase Secretion Assay of BMSCs and SFs

**Figure 5:**
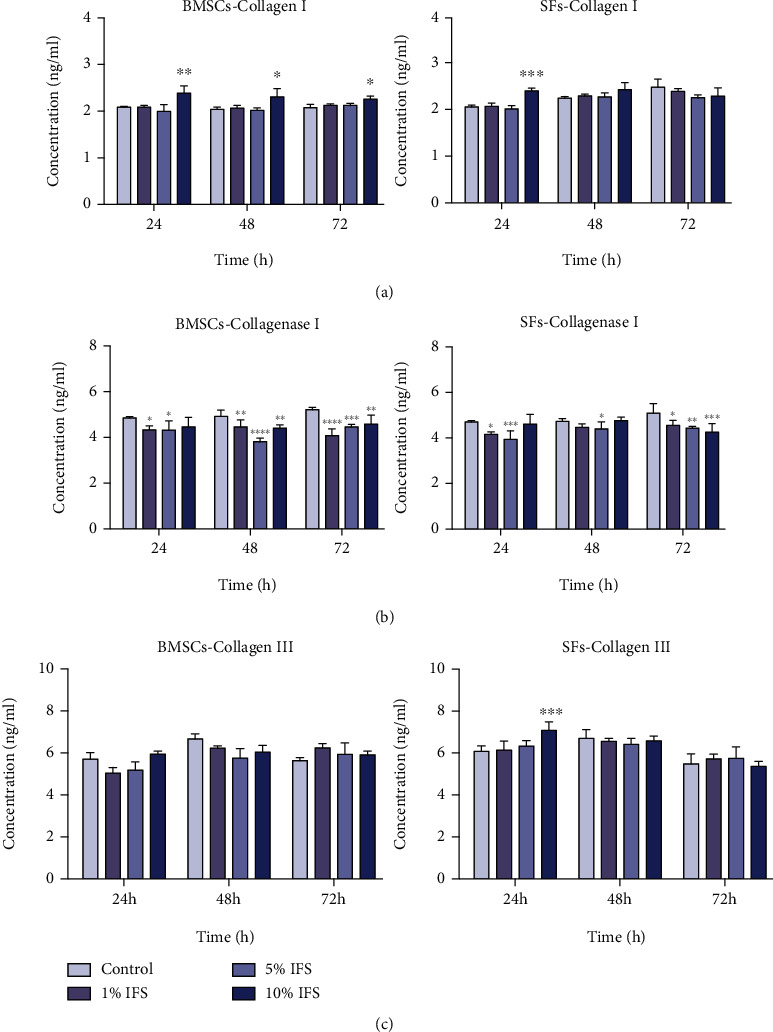
Release profiles of collagen I (a), collagenase I (b), and collagen III (c) by BMSCs and SFs after treatment with 1%, 5%, and 10% IFS for 24, 48, and 72 h (^∗^*p* < 0.05, ^∗∗^*p* < 0.01, ^∗∗∗^*p* < 0.005, and ^∗∗∗∗^*p* < 0.001 for the IFS-treated groups compared with the control group).

The IFS could promote the secretion of type I and III collagen by cells while reducing the secretion of type I collagenase. The secretion of collagen I, collagenase I, and collagen III by BMSCs and SFs at each time point is shown in Figure 5. Over the whole observation period, 10% IFS was found to have promoted the secretion of collagen I by BMSCs. After 24 h of treatment, 10% IFS was also found to have significantly promoted the secretion of collagen I by SFs. Interestingly, after treatment with IFS, the secretion of type I collagenase by both types of cells decreased significantly. In the 72 h SF group, the inhibition of type I collagenase was found to be concentration-dependent. The secretion of type III collagen by SFs was significantly promoted after treatment with IFS for 24 h.

### 3.6. Gene Expression

**Figure 6:**
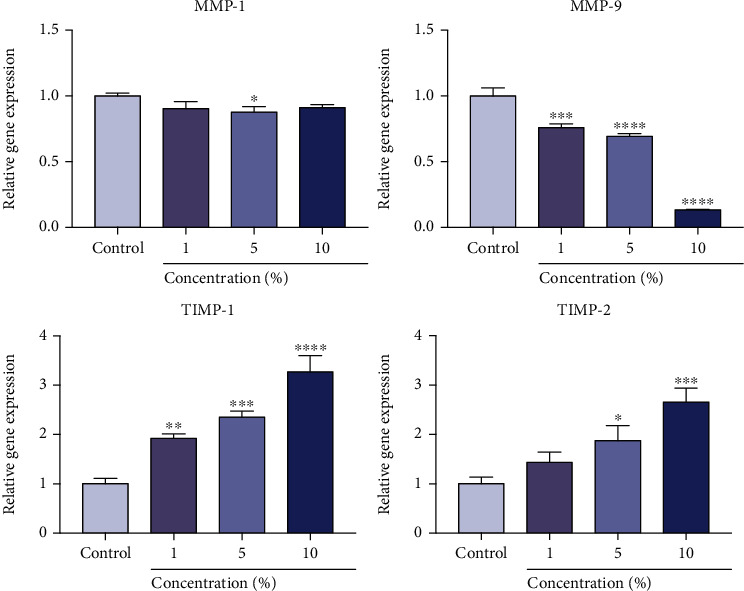
PCR analysis of the expression of genes associated with wound healing in fibroblasts cultured with IFS. (^∗^*p* < 0.05, ^∗∗^*p* < 0.01, ^∗∗∗^*p* < 0.005, and ^∗∗∗∗^*p* < 0.001 for the IFS-treated groups compared with the control group).

A quantitative real-time PCR assay of wound-healing-related genes was performed on SFs cultured with different concentrations (1%, 5%, and 10%) of IFS for 48 h (Figure 6). The expression of *MMP-1* was slightly downregulated in the 5% IFS group as compared with the control group. Importantly, the IFS could inhibit the expression of *MMP-9*, as we found significant differences between the 1%, 5%, and 10% IFS groups and the control group. In contrast to the trend of inhibition of *MMP-1* and *MMP-9*, the results showed that IFS could enhance the expression of *TIMP-1* and *TIMP-2* which are inhibitors of *MMPs*. The expression of *TIMP-1* was elevated in all IFS groups as compared with the control group, with the 10% IFS group possessing the highest value (*p* < 0.001). In addition, a significantly enhanced level of expression of *TIMP-2* was observed in the 5% and 10% IFS groups.

### 3.7. Repair and Detection of Skin Defects

**Figure 7:**
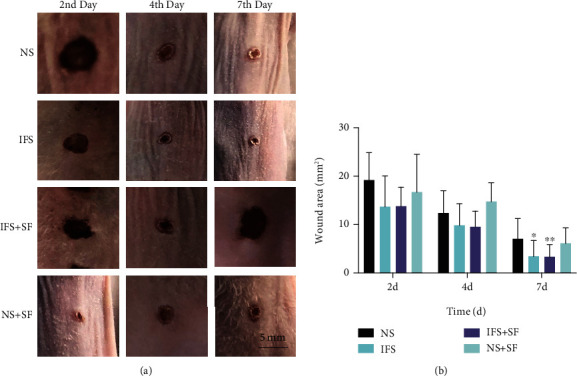
Healing of full-thickness skin wounds in nude mice. Representative images (a) and wound area of skin defects (b) at 2, 4, and 7 days after surgery (^∗^*p* < 0.05 and ^∗∗^*p* < 0.01 for the IFS, IFS + SF, and NS + SF groups compared with the NS group).

We further examined whether the IFS can promote the healing of skin defects in nude mice. The wound area was recorded macroscopically (Figure 7(a)). The experimental results show that the defect area of the IFS + SF group was significantly smaller than that of the control group on the second day after surgery (Figure 7(b)). On the 7th day after surgery, the skin defects in the mice in both the IFS + SF group and the IFS group had basically healed. The defect area of the IFS + SF group and the IFS group was significantly smaller than that of the control group. However, no significant difference was found between these two groups.

**Figure 8:**
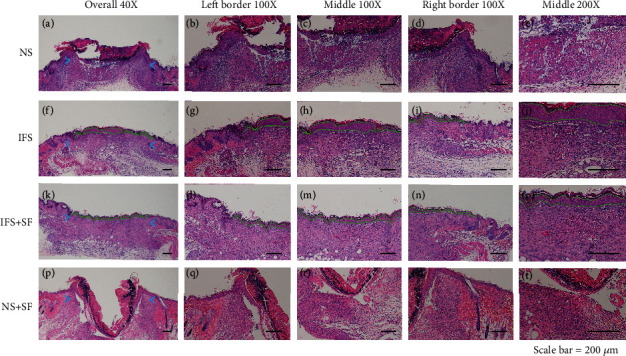
Representative HE staining sections showing the healing wound tissues of the NS, IFS, IFS + SF, and NS + SF groups 7 days after surgery. The green dotted lines indicate the stratum spinosum border. Scale bars, 200 *μ*m.

**Figure 9:**
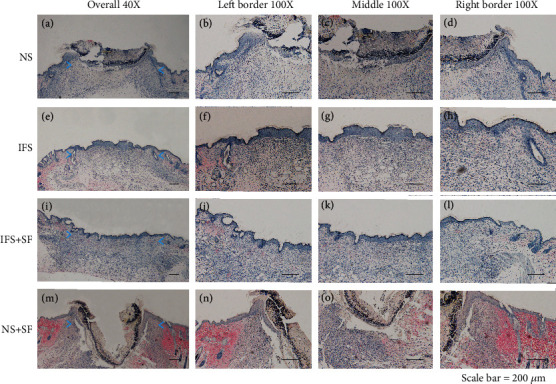
Representative Masson's trichrome staining sections showing the healing wound tissues of the NS, IFS, IFS + SF, and NS + SF groups 7 days after surgery. In Masson's trichrome staining, collagen is stained blue, cytoplasm and keratin are stained red, and nuclei are stained black. Scale bars, 200 *μ*m.

In the histological analysis, HE and Masson's trichrome staining showed that the IFS promoted the repair of the defect area (Figures 8 and 9). In both the IFS group and the IFS + SF group, the surfaces of the defect area were covered by new skin with a relatively flat new epidermis and a clear skin structure. Masson's trichrome staining showed that the collagen fibers in the IFS group and the IFS + SF group were numerous and arranged in a regular direction with the fiber bundles more parallel to the skin surface than those in the other two groups (Figure 9). The skin of mice in the IFS + SF group was close to normal skin, while the stratum spinosum of mice in the IFS group was thicker than that of mice in the IFS + SF group. This suggests that mice in the IFS group remained in the healing stage with vigorous synthesis. There were no obvious boundaries between the new skin and the surrounding tissue in the IFS and IFS + SF groups. There was an abundance of blood vessels under the new tissue in these two groups.

In the NS group, the crust was thick, and the reepithelialized junction bulged with the infiltration of inflammation (Figure 8). The stratum basale in the central part of the healing defect area was loosely arranged and disorganized without obvious epithelial pegs. Masson's trichrome staining showed that the collagen fibers were in a loose and irregular formation (Figure 9).

In the NS + SF group, the crust was thick and pitted. The surface of the healing area resembled a deep dimple. The reepithelialized connection structure was loose (Figures 8 and 9). The epidermis of the central part was loose and sometimes even separated from the subdermal part.

**Figure 10:**
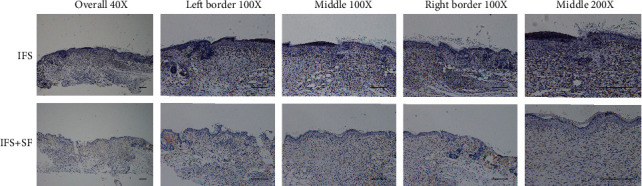
Expression of COL-I at wound sites of nude mice in the IFS and IFS + SF groups 7 days after surgery, as revealed by immunohistochemical examination. Scale bars, 200 *μ*m.

**Figure 11:**
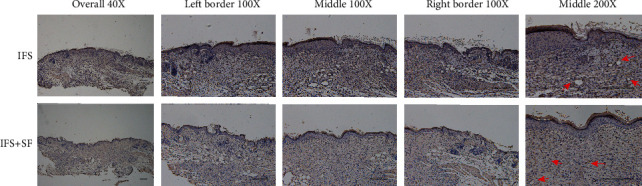
Expression of VEGF at wound sites of nude mice in the IFS and IFS + SF groups 7 days after surgery, as revealed by immunohistochemical examination. The high-magnification images show a high density of microvessels (e, j). Scale bars, 200 *μ*m.

Seven days after surgery, complete epithelialization was found in both the IFS group and the IFS + SF group. COL-I immunohistochemical images showed that both the IFS group and the IFS + SF group had a positive expression of COL-I, and the IFS was more strongly stained in these two groups (Figure 10), showing the high synthetic activity of collagen I in dermal tissue. In addition, the level of VEGF expression in the IFS group and the IFS + SF group was significantly higher than that in the other two groups. The high-magnification images displayed a high density of microvessels, suggesting enhanced angiogenesis (Figure 11).

## 4. Discussion

In this study, we obtained an IFS rich in growth factors using a simple one-step centrifugation method. We showed that this IFS promotes reepithelialization and increases vascularization in a full-thickness skin defect model. To date, numerous strategies that aim to accelerate the wound-healing process have been utilized, and different methods for the induction and acceleration of healing have been studied, such as cell therapy, bioactive therapeutic delivery, and biomaterial-based approaches [16]. The IFS, with its loose fibrin network structure, can trap a large number of white blood cells and platelets and can continuously produce and secrete growth factors. In addition, its uncrosslinked liquid scaffold structure may be conducive to the diffusion of small molecular components and can maintain the secretion of a high concentration of growth factors for at least two weeks, which is an important period for skin defect healing.

The prepared IFS demonstrated good biocompatibility and can promote cell proliferation, both of which are very important for skin regeneration [17, 18]. Interestingly, we found that the IFS can promote the secretion of both type I and III collagen, which are the staple collagen components of the extracellular matrix [16], and inhibit the secretion of collagenase to reduce the rate of degradation of collagen. We further studied the matrix metalloproteinases (*MMPs*) and tissue inhibitor metalloproteinases (*TIMPs*), which are the key enzyme systems involved in extracellular matrix (ECM) degradation and remodeling in tissue regeneration. *MMP-1* and *MMP-9* are the representative collagenase and gelatinase, respectively, for the degradation of collagen fibers. The IFS was found to simultaneously significantly reduce the expression of these two *MMPs* and significantly reduce the expression of *TIMP-1* and *TIMP-2*, which inhibit the expression of *MMPs*. It was revealed that the IFS can promote the secretion of collagen into the ECM and inhibit its degradation. This indicates that the prepared IFS has great potential in skin regeneration.

In the experiment to evaluate the repair of full-thickness skin defects on the dorsum of nude mice, wound healing was found to be promoted in both the IFS group and the IFS+SF group, and there was no significant difference between the two groups. In fact, the IFS alone could promote the healing of skin wounds. After an injury to the skin, cells migrate from the surrounding area to the wound area and regenerate the lost tissue. IFS can promote the migration, proliferation, and secretion into the matrix of stem cells and skin fibroblasts located around the defect, due to its stable and sustained release of such growth factors as PDGF, VEGF, TGF-*β*, IGF, FGF, and EGF [16, 19, 20]. For instance, EGF stimulates the migration and proliferation of fibroblasts [21] and accelerates epidermal regeneration in partial-thickness injuries [22], TGF-*β* drives collagen deposition [23], and PDGF accelerates the maturation of collagen chains [24]. Moreover, FGF is related to the downregulation of type-I procollagen gene expression, which limits the deposition of collagen by keloid fibroblasts and prevents keloid formation [25, 26]. During the 7-day observation period of this experiment, the secretion of growth factors by IFS remained at a high level. This might accelerate skin regeneration through epithelial coverage, matrix deposition, and angiogenesis. When attached to the surface of the skin defect, the IFS was found to match and fill the defects well. The collagen fiber network of the IFS could provide temporary space for the deposition of cells into the ECM and also guide the migration of surrounding cells toward the defect.

The ECM plays an important role during the repair of skin wounds as it serves as a support structure for cell proliferation, migration, and differentiation [27]. The ECM is a dynamic structure that is continuously reshaped to achieve tissue homeostasis [16]. Topically, the histological analysis showed that sections of the regenerated dermal tissue in the wound area in the IFS group and the IFS+SF group were similar to the normal tissue. The regenerated tissue of the other groups was relatively immature, and the continued remodeling of the surface area had characteristics of excessive scarring, brittleness, and/or out-of-flatness, suggesting that tissue remodeling had not been completed. This observation is consistent with the finding of lower *MMP* expression and higher *TIMP* expression in SFs treated with IFS in vitro.

Neovascularization is also a crucial step in the wound-healing process [28–32]. The formation of new blood vessels is necessary to sustain the newly formed and granulated tissue and the survival of keratinocytes. A large number of newborn blood vessels could be seen in the middle part of the healing defect area in the IFS and IFS+SF groups. Immunohistochemistry of the wound tissue revealed that the angiogenic growth factors contained in the IFS could also significantly promote angiogenesis in vivo. Neovascularization can promote the accumulation of undifferentiated mesenchymal cells in the defect area. These cells participate in the repair of defects and the reconstruction of new tissue to help form more mature skin tissue. In addition, leukocytes in the IFS are crucial to tissue regeneration as they direct and recruit cells of various types during the wound-healing process [33].

## 5. Conclusions

In summary, an IFS was extracted by one-step centrifugation. This method is relatively simple to apply and produces an easy-to-use platelet concentrate in a liquid formulation. The liquid scaffold contains white blood cells and platelets that can sustain the release of growth factors. The IFS can accelerate wound healing in nude mice full-thickness skin wounds model by promoting the migration of fibroblasts to the ECM and neovascularization at wound sites. Thus, the IFS may be utilized as a therapeutic agent alone or in combination with other biomaterials to promote tissue regeneration.

## Figures and Tables

**Table 1:** The primer sequences used in qPCR.

Gene	Sequences	
	Forward (5′–3′)	Reverse (5′–3′)
*MMP-1*	TGCTGCTACTGCTCTG	TGGAGTGAGGACGAAC
*MMP-9*	CGAGTTTCCGTTCATCTTC	ACCACCGTGGAGTCAGC
*TIMP-1*	GCTCCAGAAGTCAATCATAC	TTCCGCAGTTGTCCAG
*TIMP-2*	TGAGCAGCACGCAGAA	CCAGTCCATCCAGAGGC

## Data Availability

The data used to support the findings of this study are available from the corresponding author upon request.

## References

[1] Discher D. E., Mooney D. J., Zandstra P. W. (2009). Growth factors, matrices, and forces combine and control stem cells. *Science*.

[2] Barrientos S., Stojadinovic O., Golinko M. S., Brem H., Tomic-Canic M. (2008). Growth factors and cytokines in wound healing. *Wound Repair and Regeneration*.

[3] Atashi F., Jaconi M. E., Pittet-Cuenod B., Modarressi A. (2015). Autologous platelet-rich plasma: a biological supplement to enhance adipose-derived mesenchymal stem cell expansion. *Tissue Engineering. Part C, Methods*.

[4] Masuki H., Okudera T., Watanebe T. (2016). Growth factor and pro-inflammatory cytokine contents in platelet-rich plasma (PRP), plasma rich in growth factors (PRGF), advanced platelet-rich fibrin (A-PRF), and concentrated growth factors (CGF). *International journal of implant dentistry*.

[5] Bielecki T., Ehrenfest D. M. D. (2012). Platelet-rich plasma (PRP) and platelet-rich fibrin (PRF): surgical adjuvants, preparations for in situ regenerative medicine and tools for tissue engineering. *Current Pharmaceutical Biotechnology*.

[6] Choukroun J., Diss A., Simonpieri A. (2006). Platelet-rich fibrin (PRF): a second-generation platelet concentrate. Part V: histologic evaluations of PRF effects on bone allograft maturation in sinus lift. *Oral Surgery, Oral Medicine, Oral Pathology, Oral Radiology, and Endodontics*.

[7] Dohan D. M., Choukroun J., Diss A. (2006). Platelet-rich fibrin (PRF): a second-generation platelet concentrate. Part II: platelet-related biologic features. *Oral Surgery, Oral Medicine, Oral Pathology, Oral Radiology, and Endodontics*.

[8] Dohan D. M., Choukroun J., Diss A. (2006). Platelet-rich fibrin (PRF): a second-generation platelet concentrate. Part I: technological concepts and evolution. *Oral Surgery, Oral Medicine, Oral Pathology, Oral Radiology, and Endodontics*.

[9] Sammartino G., Ehrenfest D. M. D., Carile F., Tia M., Bucci P. (2011). Prevention of hemorrhagic complications after dental extractions into open heart surgery patients under anticoagulant therapy: the use of leukocyte- and platelet-rich fibrin. *Journal of oral implantology*.

[10] Hoaglin D. R., Lines G. K. (2013). Prevention of localized osteitis in mandibular third-molar sites using platelet-rich fibrin. *International journal of dentistry*.

[11] Yelamali T., Saikrishna D. (2015). Role of platelet rich fibrin and platelet rich plasma in wound healing of extracted third molar sockets: a comparative study. *Journal of maxillofacial and oral surgery*.

[12] Jankovic S., Aleksic Z., Klokkevold P. (2012). Use of platelet-rich fibrin membrane following treatment of gingival recession: a randomized clinical trial. *International Journal of Periodontics and Restorative Dentistry*.

[13] Ghanaati S., Booms P., Orlowska A. (2014). Advanced platelet-rich fibrin: a new concept for cell-based tissue engineering by means of inflammatory cells. *Journal of Oral Implantology*.

[14] Honda H., Tamai N., Naka N., Yoshikawa H., Myoui A. (2013). Bone tissue engineering with bone marrow-derived stromal cells integrated with concentrated growth factor in *Rattus norvegicus* calvaria defect model. *Journal of Artificial Organs*.

[15] Miron R. J., Fujioka-Kobayashi M., Hernandez M. (2017). Injectable platelet rich fibrin (i-PRF): opportunities in regenerative dentistry?. *Clinical Oral Investigations*.

[16] Nour S., Baheiraei N., Imani R. (2019). A review of accelerated wound healing approaches: biomaterial- assisted tissue remodeling. *Journal of Materials Science. Materials in Medicine*.

[17] Brucoli M., Sonzini R., Bosetti M., Boffano P., Benech A. (2018). Plasma rich in growth factors (PRGF) for the promotion of bone cell proliferation and tissue regeneration. *Oral and Maxillofacial Surgery*.

[18] Brini A. T., Ceci C., Taschieri S. (2016). Effect of an activated platelet concentrate on differentiated cells involved in tissue healing. *The Journal of Craniofacial Surgery*.

[19] Laiva A. L., O’Brien F. J., Keogh M. B. (2018). Innovations in gene and growth factor delivery systems for diabetic wound healing. *Journal of Tissue Engineering and Regenerative Medicine*.

[20] Yildirimer L., Thanh N. T., Seifalian A. M. (2012). Skin regeneration scaffolds: a multimodal bottom-up approach. *Trends in Biotechnology*.

[21] Nishida T., Tanaka H., Nakagawa S., Sasabe T., Awata T., Manabe R. (1984). Fibronectin synthesis by the rabbit cornea: effects of mouse epidermal growth factor and cyclic AMP analogs. *Japanese Journal of Ophthalmology*.

[22] Brown G. L., Curtsinger L., Brightwell J. R. (1986). Enhancement of epidermal regeneration by biosynthetic epidermal growth factor. *The Journal of Experimental Medicine*.

[23] Jun J. I., Lau L. F. (2018). Resolution of organ fibrosis. *The Journal of Clinical Investigation*.

[24] Mihaylova Z., Tsikandelova R., Sanimirov P., Gateva N., Mitev V., Ishkitiev N. (2018). Role of PDGF-BB in proliferation, differentiation and maintaining stem cell properties of PDL cells _*in vitro*_. *Archives of Oral Biology*.

[25] Shi H. X., Lin C., Lin B. B. (2013). The anti-scar effects of basic fibroblast growth factor on the wound repair *in vitro* and *in vivo*. *PLoS One*.

[26] Xie J. L., Bian H. N., Qi S. H. (2008). Basic fibroblast growth factor (bFGF) alleviates the scar of the rabbit ear model in wound healing. *Wound Repair and Regeneration*.

[27] Schultz G. S., Wysocki A. (2005). Extracellular matrix: review of its roles in acute and chronic wounds. *World wide wounds*.

[28] Martin P. (1997). Wound healing—aiming for perfect skin regeneration. *Science*.

[29] Falanga V. (2005). Wound healing and its impairment in the diabetic foot. *Lancet*.

[30] Singer A. J., Clark R. A. (1999). Cutaneous wound healing. *The New England Journal of Medicine*.

[31] Rousselle P., Braye F., Dayan G. (2019). Re-epithelialization of adult skin wounds: cellular mechanisms and therapeutic strategies. *Advanced Drug Delivery Reviews*.

[32] Tonnesen M. G., Feng X., Clark R. A. (2000). Angiogenesis in wound healing. *Journal of Investigative Dermatology Symposium Proceedings*.

[33] Bielecki T., Ehrenfest D. M. D., Everts P. A., Wiczkowski A. (2012). The role of leukocytes from L-PRP/L-PRF in wound healing and immune defense: new perspectives. *Current Pharmaceutical Biotechnology*.

